# Limited Association Between Body Mass Index and Selected Components of Physical Fitness in Higher Education Physical Education Students: A Sex- and Country-Specific Analysis

**DOI:** 10.3390/sports14050167

**Published:** 2026-04-22

**Authors:** Agnieszka Wasiluk, Viktoriia Kyrychenko, Grațiela-Flavia Deak, Robert Wilczewski

**Affiliations:** 1 Faculty of Physical Education and Health in Biala Podlaska, Jozef Pilsudski University of Physical Education in Warsaw, 00-968 Warsaw, Poland; robert.wilczewski@awf.edu.pl; 2 Interdisciplinary Research Center in the Domain of Physical Education and Sport, Babeș-Bolyai University, 400084 Cluj-Napoca, Romania; victoria.kyrychenko@ubbcluj.ro; 3 Department of Kinesiotherapy and Theoretical Disciplines, Babeș-Bolyai University, 400084 Cluj-Napoca, Romania; gratiela.deak@ubbcluj.ro

**Keywords:** body mass index, physical fitness, physical education students, Eurofit test battery, sex differences, cross-sectional study

## Abstract

Background: Body mass index (BMI) is widely used as a simple anthropometric indicator, but its functional relevance to physical fitness in physically active populations, such as Physical Education students, remains debated. Aim: This study examined the association between BMI and selected components of physical fitness in Physical Education students, considering sex and country differences. Methods: A cross-sectional study was conducted among undergraduate Physical Education students from Poland and Romania (*n* = 515; mean age: 21.64 ± 1.34 years). BMI was calculated from measured height and body mass and analyzed as both a continuous and categorical variable. Physical fitness was assessed using three Eurofit tests evaluating upper-limb movement speed, trunk muscular endurance, and lower-limb explosive power. Analyses included correlation methods and multiple linear regression models with subgroup analyses, interaction terms, and quadratic BMI terms to assess nonlinearity. Results: Associations between BMI and fitness components were small in magnitude and inconsistent (r = −0.28 to 0.143; β = −1.614 to 0.005) and varied across tests and subgroups. No significant interaction effects by sex or country were observed, as interaction terms were not statistically significant, and no clear nonlinear relationships were identified. Sex and country were significantly associated with performance levels, whereas BMI contributed only marginally to explaining variability (ΔR^2^ = 0.005–0.011). Conclusions: BMI showed limited and inconsistent associations with the assessed fitness components in this relatively homogeneous group of Physical Education students. It should be interpreted cautiously as a functional indicator and complemented with more precise measures of body composition and physical fitness.

## 1. Introduction

Body mass index (BMI) is one of the most widely used anthropometric indicators in population-based research, primarily due to its simplicity and suitability for international comparisons. At the same time, numerous studies have highlighted its limitations in assessing obesity and health risk, resulting from the lack of information on body composition and fat distribution [1,2,3]. Despite these limitations, BMI remains commonly applied in sport and exercise sciences, particularly in studies of physical fitness, where body mass relative to height may influence the performance of specific motor tasks. Previous research indicates that the relationships between BMI and physical fitness are heterogeneous and depend on both the fitness component assessed and the characteristics of the studied population [4,5]. These relationships may be explained by biomechanical and physiological mechanisms. In tasks requiring body mass displacement or relative force production, higher BMI may increase mechanical load and reduce performance, particularly when associated with higher fat mass [2,3]. In contrast, in tasks with minimal body mass involvement, BMI may have limited functional impact. Differences in body composition at similar BMI values may further modify these associations [1,3]. Among student populations, BMI has been shown to be selectively associated with fitness test performance, with the direction and strength of these associations differing across components such as strength, endurance, and speed [6]. Physical fitness is a multidimensional construct that includes components such as cardiorespiratory endurance, muscular strength, muscular endurance, flexibility, speed, and coordination. However, in the present study, only selected components of physical fitness were assessed, specifically upper-limb movement speed, trunk muscular endurance, and lower-limb explosive power.

The role of BMI in relation to physical fitness is further modified by sex. Women and men differ in body composition and in the proportion of muscle and fat mass, meaning that the same BMI value may have different functional implications [7,8]. This can be explained by physiological and biomechanical factors. Men typically have higher muscle mass and greater absolute and relative strength, whereas women generally have a higher proportion of body fat, which may influence performance in tasks requiring body mass displacement. In addition, neuromuscular characteristics and differences in body size and scaling may modify the relationship between BMI and physical fitness [7,8]. Furthermore, the observed relationships may be influenced by country-specific factors, such as educational systems, habitual physical activity levels, and cultural conditions [9,10]. In the present study, country was treated as a contextual variable that may indirectly reflect differences in training exposure, lifestyle, and environmental factors. Therefore, it was hypothesized that country would act as a moderator of the relationship between BMI and physical fitness, reflecting potential differences in training exposure, lifestyle, and environmental conditions; however, no a priori directional hypothesis was formulated.

Students of Physical Education constitute a particularly relevant study group. As future professionals in health promotion and physical activity, they are characterized by relatively high activity levels and by homogeneity in age and educational profile, which facilitates a more controlled analysis of relationships between somatic characteristics and physical fitness [11,12,13]. Despite the growing body of research on BMI and physical fitness, relatively few studies have directly examined these associations while simultaneously accounting for sex and country context in populations of Physical Education students. Therefore, the present study was designed as a replication and verification analysis aimed at assessing the stability of previously reported relationships between BMI and physical fitness in a homogeneous population of students enrolled in Physical Education programs, which include regular practical classes [6,14]. In this context, replication refers to re-examining previously reported BMI–fitness associations in a comparable population, whereas stability was understood as the consistency of the direction and magnitude of these relationships across subgroups (sex and country) and different analytical approaches. Although the literature suggests the possible presence of nonlinear relationships between BMI and physical fitness, both linear and potential nonlinear components of this association were considered to provide a more comprehensive evaluation of the BMI–fitness component relationships in comparable student populations. Such relationships may take the form of U- or J-shaped associations, meaning that both low and high BMI values may be associated with reduced levels of physical fitness [2,14]. This pattern may be explained by underlying physiological mechanisms, as both high BMI (associated with excess fat mass and increased mechanical load) and low BMI (potentially reflecting lower muscle mass) may negatively affect physical performance. However, it should be noted that detecting such nonlinear relationships typically requires larger sample sizes and sufficient variability, which may limit the sensitivity of analyses conducted in relatively homogeneous populations. Therefore, approaches based solely on continuous variables may not fully capture potential threshold effects, which supports the consideration of categorical approaches. To address these issues, BMI was analyzed using both continuous and categorical approaches.

Despite the growing body of literature on the relationship between BMI and physical fitness, evidence regarding its functional relevance in physically active populations remains inconsistent, particularly in young adults engaged in regular physical training. Moreover, few studies have simultaneously examined these relationships while accounting for both sex- and country-related differences, which may reflect biological variability as well as differences in educational systems and physical activity contexts. Therefore, the present study aimed to address this gap by investigating the associations between BMI and selected components of physical fitness in Physical Education students from two European countries.

This study aimed to examine the association between BMI and selected components of physical fitness in Physical Education students, considering sex- and country-related differences, and to assess the stability of these relationships across subgroups and analytical approaches.

## 2. Materials and Methods

### 2.1. Participants

The study employed a cross-sectional design and involved undergraduate Physical Education students recruited from two academic institutions: the Faculty of Physical Education and Health in Biała Podlaska, Poland, and the Faculty of Physical Education and Sport of Babeș-Bolyai University in Cluj-Napoca, Romania. The final analytical sample consisted of 258 students from Poland (180 men and 78 women) and 257 students from Romania (170 men and 87 women). All participants were full-time Physical Education students and regularly attended practical classes included in the study curriculum. Inclusion criteria comprised the absence of self-reported metabolic or cardiovascular diseases and the ability to complete the full physical fitness testing protocol. Participants’ age was recorded for descriptive purposes only. Due to the relatively narrow age range of the study population (mean age: 21.6 ± 1.34 years) and the homogeneous educational and training profile of the participants, age was not included as a covariate in the main analyses. Detailed age characteristics, including mean values and variability across subgroups, are presented in Table 1, allowing readers to assess the variability of this variable. This decision was consistent with the adopted analytical framework, which focused on evaluating differences in the associations between body mass index (BMI) and selected components of physical fitness with respect to sex and country. It should also be noted that detailed information regarding habitual physical activity levels, sport specialization, or training experience was not collected and therefore could not be included in the analyses.

### 2.2. Anthropometric Measurements

Anthropometric measurements were conducted in accordance with standardized procedures recommended by the International Society for the Advancement of Kinanthropometry (ISAK) [15]. Body height was measured to the nearest 0.1 cm using a portable stadiometer, and body mass was assessed to the nearest 0.1 kg using a calibrated electronic scale. All measurements were taken barefoot, with participants wearing light clothing. Body mass index (BMI) was calculated as body mass (kg) divided by height squared (m^2^) and treated primarily as a continuous variable, with additional categorical analyses performed to assess potential threshold effects.

### 2.3. Physical Fitness Assessment

Physical fitness was assessed using three standardized tests from the Eurofit test battery [16], selected to evaluate specific components of physical fitness relevant to Physical Education students, namely upper-limb movement speed, trunk muscular endurance, and lower-limb explosive power. The plate tapping test was used to assess upper-limb movement speed and coordination, the sit-up test to evaluate trunk muscular endurance, and the standing long jump test to measure lower-limb explosive power. These tests are widely used in physical fitness assessment and have been demonstrated to be valid and reliable measures of the respective fitness components, as supported by previous studies and systematic reviews [16,17]. The applied test battery allowed for the assessment of fitness components differing in functional and biomechanical demands, particularly with respect to the role of body mass during task execution. Tests requiring displacement of total body mass or the generation of relative power output (sit-ups and standing long jump) may be more sensitive to differences in relative body mass and thus potentially associated with BMI. In particular, the standing long jump reflects lower-limb relative power output, whereas the sit-up test involves repeated lifting of body mass, reflecting relative muscular endurance. In contrast, the plate tapping test, which primarily reflects upper-limb movement speed and coordination, involves minimal body mass loading, supporting its lower sensitivity to BMI-related variability. All tests were conducted under standardized conditions and supervised by experienced examiners to ensure compliance with established testing protocols. It should be emphasized that the applied test battery allows conclusions to be drawn only with regard to selected components of physical fitness—namely movement speed, muscular endurance, and explosive power—and does not reflect physical fitness as a multidimensional construct.

### 2.4. Statistical Analysis

All data were entered into a secure database and analyzed using Statistica version 13.0 (StatSoft, Kraków, Poland). Continuous variables are presented as means and standard deviations. Analyses were performed separately in four subgroups defined by sex and country: Polish men, Polish women, Romanian men, and Romanian women. This approach allowed for the assessment of whether associations between body mass index (BMI) and physical fitness test outcomes differed according to sex and country context.

The analytical strategy followed a structured, hypothesis-informed approach and should be interpreted as exploratory rather than strictly confirmatory. Correlation analyses were treated as exploratory, while multiple regression models were used to further examine these relationships. In the first stage, associations between BMI and performance in individual fitness tests were examined using Pearson’s correlation coefficient, calculated separately within each subgroup. These analyses served as an initial assessment of the direction and strength of linear relationships between BMI and selected fitness components. Therefore, no formal corrections for multiple comparisons were applied, as the analyses were intended to explore potential patterns of association. Correlation results were interpreted as hypothesis-generating, taking into account their auxiliary role relative to subsequent analyses. Given the number of comparisons performed, the risk of Type I error cannot be excluded, and the findings require confirmation in future studies using corrected analytical approaches. Therefore, the results should be interpreted with caution.

The use of Pearson’s correlation was justified by the continuous nature of the analyzed variables and the study aim of assessing the linear component of the BMI–fitness relationships. To complement the continuous approach and to better capture potential threshold effects, BMI was additionally analyzed as a categorical variable. Participants were initially classified according to standard WHO BMI classification criteria (underweight, normal weight, overweight, obese) [18]. However, due to the low number of participants in the underweight and obese categories, BMI groups were collapsed into two categories: non-overweight (BMI < 25 kg/m^2^) and overweight/obese (BMI ≥ 25 kg/m^2^). Differences in physical fitness outcomes between these groups were assessed using independent samples t-tests, conducted separately within each sex–country subgroup. In the subsequent stage, multiple linear regression models were applied to evaluate whether the associations between BMI and fitness test outcomes differed by sex or country. These models simultaneously included BMI, sex, and country as predictors of fitness performance and allowed for testing interactions between BMI and sex as well as between BMI and country. Additional regression analyses were also conducted separately within each subgroup to facilitate more detailed interpretation of observed associations and were intended to support interpretation rather than serve as independent confirmatory analyses.

To formally assess potential nonlinear relationships between BMI and fitness test outcomes, a quadratic BMI term (BMI^2^) was additionally included in the regression models. Prior to computing the quadratic term, BMI was centered around the sample mean to reduce multicollinearity between linear and quadratic terms. Statistical significance of the BMI^2^ term was interpreted as evidence of a curvilinear relationship between BMI and the analyzed fitness outcome. Assumptions of linear regression models, including linearity, homoscedasticity, normality of residuals, and the presence of influential observations, were examined and revealed no substantial violations. Statistical analyses were conducted separately for each fitness test outcome, without direct comparisons between tests with different scales and characteristics; therefore, no standardization of outcomes was applied. All statistical tests were two-tailed, and statistical significance was set at *p* < 0.05. Additionally, the potential influence of age was examined in supplementary analyses to verify its relevance as a covariate, although it was not included in the main models due to the narrow age range of the sample.

## 3. Results

### 3.1. Descriptive Characteristics of the Study Sample

Descriptive statistics for anthropometric variables and physical fitness test outcomes, stratified by sex and country of origin, are presented in Table 1. Clear differences were observed both between women and men and between students from Poland and Romania with respect to somatic characteristics and performance in the selected physical fitness tests assessed in the study.

### 3.2. Associations Between BMI and Physical Fitness: Correlation Analysis

The correlation analysis indicated that associations between body mass index (BMI) and performance in selected physical fitness tests were selective and limited to individual tests and specific student subgroups (Table 2). In the plate tapping test, no significant associations between BMI and performance were observed in either women or men, regardless of country of origin. For tests assessing muscular endurance and lower-limb power, isolated significant correlations were identified. In the sit-up test, higher BMI values were associated with poorer performance only among women from Poland and men from Romania, whereas in the remaining subgroups these associations did not reach statistical significance. Similarly, in the standing long jump test, a significant negative correlation between BMI and jump distance was observed exclusively among Polish men. It should be emphasized, however, that the statistically significant correlations identified were of small magnitude, lacked consistency across the analyzed subgroups, and did not form a coherent pattern of associations. The observed relationships were small and inconsistent, indicating limited practical relevance, particularly for isolated statistically significant associations.

### 3.3. Comparison of Physical Fitness Between BMI Categories

The comparison of physical fitness test results between the identified BMI categories within sex- and country-defined subgroups is presented in Table 3. It was observed that differences between BMI categories were limited and inconsistent across the analyzed groups and fitness components. In particular, no statistically significant differences between BMI categories were found in any subgroup in the plate tapping test. With regard to the sit-up test, significantly lower performance in the BMI ≥ 25 kg/m^2^ group was observed only among Romanian men, while no significant differences were found in the remaining subgroups. A similar selective pattern was observed in the standing long jump test, where participants with BMI ≥ 25 kg/m^2^ achieved significantly poorer results compared with those with BMI < 25 kg/m^2^, among Polish men and women. In the remaining subgroups, no significant differences were observed. Potential threshold effects related to BMI categories were limited and depended on both the specific component of physical fitness and the subgroup analyzed.

### 3.4. Regression Analyses: Sex- and Country-Related Variation in the BMI–Fitness Component Relationships

The results of multiple linear regression analyses examining whether the relationship between body mass index (BMI) and performance in selected physical fitness tests differed according to sex and country are presented in Table 4. The models included BMI, sex, and country, as well as interaction terms between BMI and sex and between BMI and country, and additionally assessed potential non-linear relationships by including a quadratic BMI term (BMI^2^). The analyses did not show significant associations between BMI and plate tapping or sit-up performance, whereas a small but statistically significant negative association was observed for long jump, indicating that higher BMI was associated with slightly lower performance. At the same time, no significant interactions between BMI and sex or between BMI and country were found, although a borderline interaction with country was observed, suggesting no evidence that the nature of the BMI–performance relationship differed across these factors. The quadratic BMI term was not statistically significant in any of the models, indicating no clear evidence of non-linear relationships between BMI and the analyzed fitness outcomes (Table S1), although more complex non-linear patterns cannot be completely excluded. Independent of BMI, significant differences in performance levels were observed according to sex and country. Men achieved better results in strength- and power-oriented tests, whereas students from Romania performed better in the plate tapping test and worse in strength- and power-oriented tests compared with students from Poland. These differences were not significantly modified by BMI and may be influenced by other factors such as physical activity levels, training experience, or sport specialization. The absence of correction for multiple comparisons may increase the risk of Type I error and should be considered when interpreting the results.

The potential influence of age was also examined. Age was weakly associated with plate tapping performance (r = 0.16; *p* < 0.001), explaining approximately 2.6% of the variance. No significant associations were observed for sit-ups (r = 0.07; *p* = 0.13) or long jump (r = 0.01; *p* = 0.81). Overall, the magnitude of these associations was trivial to small, indicating that age did not meaningfully influence the interpretation of the main findings.

Model fit varied across the analyzed fitness tests (Table 5). The lowest coefficients of determination (R^2^) were observed for the plate tapping test, whereas higher values were found for the sit-up test and the standing long jump. In the standing long jump model, a small but statistically significant negative association between BMI and jump distance was observed; however, this trend was not supported by a significant quadratic BMI term and did not indicate the presence of a curvilinear relationship. It should be emphasized that the relatively higher R^2^ values observed in the regression models were largely driven by the inclusion of demographic variables, particularly sex, whereas the contribution of BMI—both linear and nonlinear—to explaining variability in physical fitness outcomes was limited. This was further supported by model comparisons, which showed that the inclusion of BMI variables increased the explained variance only marginally (ΔR^2^ = 0.005–0.011).

BMI contributed only marginally to the explained variance in the assessed components of physical fitness in the studied population of Physical Education students, indicating limited practical relevance.

## 4. Discussion

The present findings indicate that the associations between body mass index (BMI) and performance in the selected physical fitness tests assessed in this study were small in magnitude, selective, and dependent on the specific component of physical fitness analyzed, indicating limited practical relevance. In particular, a small but statistically significant association was observed in selected subgroups for lower-limb explosive power (standing long jump), whereas no consistent associations were found for the remaining fitness components. In the examined population of young, physically active adults, characterized by a relatively homogeneous BMI distribution, BMI demonstrated limited explanatory value. This is consistent with the model comparisons indicating only a marginal increase in explained variance associated with BMI. Its relevance became apparent only under specific functional conditions, primarily in tasks requiring body mass displacement or the generation of relative power. These results suggest that BMI has limited value as a functional indicator in this relatively homogeneous sample. It may be better considered as a simplified measure of body mass relative to height that does not account for key aspects of body composition, such as the proportion and distribution of muscle and fat mass. Similar conclusions have been reported in studies conducted among student populations and physically active individuals, showing that associations between BMI and the assessed fitness components are generally small and strongly dependent on the fitness component assessed [19]. Importantly, the results obtained using both continuous and categorical BMI approaches were consistent, reinforcing the conclusion that BMI–fitness associations in this population are limited in magnitude and context-dependent.

The absence of significant associations between BMI and performance in the plate tapping test across all analyzed subgroups suggests that the upper-limb speed and coordination component evaluated in this study is only minimally related to body mass. This finding is consistent with reports indicating that coordination abilities and movement regulation speed are more strongly determined by neuromotor factors, such as central nervous system processing, motor unit recruitment, and intermuscular coordination, as well as motor competence, than by anthropometric characteristics, particularly in short-duration tasks with low strength demands [20,21,22]. Similar observations have been reported in studies on motor coordination, which have demonstrated weak or nonsignificant relationships between BMI and coordination abilities, further supporting the dominant role of neuromotor mechanisms over somatic factors [23].

For tests assessing muscular endurance and strength-related capacities, no consistent associations between BMI and performance were observed across the entire sample. In the sit-up test, some subgroup-specific tendencies were observed, suggesting that in tasks requiring repeated lifting and stabilization of body mass, BMI may represent an additional functional load unless compensated by adequate relative strength. At the same time, the lack of a uniform pattern across the sample confirms the limited utility of BMI as an indicator of muscular endurance-related performance in physically active individuals and is consistent with previous studies reporting weak and inconsistent associations between BMI and the assessed components of physical fitness in this population [12,24].

The most pronounced, though still weak, associations between BMI and performance in tests assessing lower-limb explosive power were observed for the standing long jump. In the present study, a small but significant relationship was observed, with higher BMI values associated with shorter jump distances. This observation is consistent with reports indicating that in tasks requiring dynamic acceleration of total body mass, higher BMI may limit the ability to generate relative power, even among individuals with a generally high level of the assessed components of physical fitness [25,26]. At the same time, the overall pattern of results suggests that these associations are context-dependent and not consistent across all analyzed components, highlighting the heterogeneous nature of BMI–power relationships and confirming that BMI does not constitute a universal predictor of explosive power-related performance, even in physically active populations [27].

An important aspect of the present study was the formal examination of potential nonlinear relationships between BMI and the assessed components of physical fitness. The inclusion of a quadratic BMI term in the regression models did not provide evidence of significant curvilinear effects in any of the analyzed tests, indicating that no clear evidence for nonlinear BMI–fitness component relationships was found in this population of Physical Education students. It should be noted that the ability to detect nonlinear relationships may be limited by the statistical power of the analysis, and the present study may have been underpowered to detect small quadratic (non-linear) effects. This finding contrasts with reports of nonlinear BMI–fitness component relationships, such as inverted J-shaped associations observed in large student samples using quadratic models [28] and parabolic relationships reported in children and adolescents [29,30]. Therefore, the absence of statistically significant non-linear effects in the present study may reflect limited statistical power to detect such relationships, particularly in a relatively homogeneous sample such as the present cohort of Physical Education students.

To further verify potential nonlinear effects, BMI was also analyzed as a categorical variable, allowing for the assessment of threshold effects. The results of this complementary analysis indicated that differences between BMI categories were limited and inconsistent across subgroups and the assessed fitness components. Significant differences were observed only in a few analyses and did not form a consistent pattern across all analyzed groups and tests. These findings are consistent with previous reports indicating the limited ability of BMI to reflect both functional aspects of the assessed components of physical fitness and actual body composition, particularly in physically active populations, in whom BMI may lead to misclassification due to a high proportion of fat-free mass [2,3]. Taken together, the results of both continuous and categorical analyses suggest that BMI may have limited relevance in explaining the assessed components of physical fitness in the studied population and that its associations with these components are weak and context-dependent. It should also be noted that detecting non-linear relationships, including quadratic effects, typically requires larger sample sizes and greater variability than detecting linear associations. Therefore, the absence of statistically significant non-linear effects in the present study may reflect limited statistical power rather than their true absence.

Regression analyses did not provide evidence for variation in the nature of BMI–fitness component relationships according to sex or country of origin. At the same time, significant differences in absolute performance levels were observed between the analyzed groups, and these differences persisted independently of BMI values. This finding points to a greater importance of factors such as motor preparation, physical activity profile, and training experience than body mass alone, which is consistent with previous reports [9,31,32].

The strengths of this study include a relatively large sample size, the international scope of the analyses, and the use of standardized physical fitness tests. Although both continuous and categorical approaches were applied in the present study, it cannot be excluded that more flexible analytical strategies, such as spline-based models, could reveal relationships not detected by linear or quadratic models. Therefore, the conclusion regarding the limited functional utility of BMI in relation to the assessed components of physical fitness should be interpreted with caution. Limitations include the cross-sectional design, the lack of data on physical activity level, sport specialization, and training experience, and the use of BMI as the sole anthropometric indicator. The absence of these variables may introduce residual confounding, as differences in physical activity level, training experience, or sport specialization may independently influence physical fitness performance and may either mask or amplify the observed associations between BMI and fitness outcomes. The cross-sectional design, in particular, precludes causal interpretation of the observed associations. The applied test battery allows conclusions to be drawn only with respect to selected components of physical fitness. Moreover, the observed differences between students from Poland and Romania are descriptive in nature and do not allow causal inferences; rather, they should be considered a starting point for further investigation.

Considering these limitations is essential for the appropriate interpretation of the findings and for assessing their potential practical applications. In Physical Education teaching, including school-based settings, as well as in the training of future teachers and coaches, BMI should be interpreted with caution and should not be used as a standalone indicator of the assessed components of physical fitness in physically active individuals. Its interpretation should always be complemented by functional assessments of motor abilities. In diagnostic practice, this implies the need for cautious use of BMI in evaluating the assessed components of physical fitness in Physical Education students and avoiding inferences about motor abilities based solely on this indicator. Future research should incorporate more precise measures of body composition and relative strength and power, as well as longitudinal designs allowing the assessment of changes in relationships between somatic characteristics and the assessed components of physical fitness over time.

## 5. Conclusions

The present study demonstrated that body mass index (BMI) appears to have limited value in explaining performance in selected physical fitness tests in this relatively homogeneous group of Physical Education students. The observed associations were small in magnitude and selective, depending on the specific component of physical fitness analyzed. Both linear and non-linear analyses did not provide consistent evidence of a meaningful role of BMI for most assessed fitness components, suggesting that its usefulness as a functional measure in relation to the assessed components of physical fitness in this specific population of young, physically active adults may be limited. At the same time, differences in performance in the assessed fitness components were observed between sexes and between students from Poland and Romania, and these differences persisted independently of BMI values. These findings suggest a potentially greater importance of factors related to motor preparation, physical activity profile, and training experience compared with body mass alone. The results indicate the need to use more precise measures of body composition and specific components of physical fitness in research on physically active populations, as such measures may better reflect actual motor capabilities within similar populations.

## Figures and Tables

**Table 1:** Descriptive statistics (mean ± SD) for anthropometric and fitness variables by group.

Variable	Poland Male	Poland Female	Romanian Male	Romanian Female
Age [years]	22.15 ± 1.00	21.77 ± 0.93	20.97 ± 1.39	21.75 ± 1.89
Height [cm]	180.97 ± 7.38	167.17 ± 5.98	179.48 ± 6.63	165.93 ± 6.78
Weight [kg]	80.40 ± 10.89	61.27 ± 7.96	77.26 ± 12.22	59.39 ± 7.85
BMI [kg/m^2^]	24.53 ± 2.86	21.89 ± 2.36	23.91 ± 3.00	21.54 ± 2.23
Plate Tapping [s]	10.04 ± 1.20	10.16 ± 1.36	8.48 ± 0.93	9.46 ± 0.96
Sit-Ups [30 s]	31.28 ± 4.20	27.10 ± 3.46	25.16 ± 4.00	21.68 ± 3.89
Long Jump [cm]	242.01 ± 21.28	192.90 ± 18.09	231.36 ± 21.91	179.83 ± 18.51

**Table 2:** Pearson correlations between BMI and fitness tests in the four sex–country groups.

Country	Sex	Test	*n*	*r*	*p*
Poland	Male	Plate Tapping [s]	179	0.019	0.797
		Sit-ups [30 s]	179	−0.078	0.298
		Long Jump [cm]	179	−0.228	0.002
	Female	Plate Tapping [s]	78	0.03	0.795
		Sit-ups [30 s]	78	−0.28	0.013
		Long Jump [cm]	78	−0.149	0.192
Romania	Male	Plate Tapping [s]	170	0.067	0.382
		Sit-ups [30 s]	170	−0.183	0.017
		Long Jump [cm]	170	−0.058	0.454
	Female	Plate Tapping [s]	87	−0.134	0.215
		Sit-ups [30 s]	87	0.143	0.186
		Long Jump [cm]	87	−0.091	0.403

Explanations: *n*—sample size; *r*—Pearson’s correlation coefficient; *p*—level of statistical significance.

**Table 3:** Comparison of physical fitness test results between BMI categories (<25 and ≥25 kg/m^2^) across sex–country subgroups.

Group	Sex	Test	<25 (Mean ± SD)	≥25 (Mean ± SD)	*p*
Poland	Male	Plate tapping [s]	10.02 ± 1.22	10.12 ± 1.19	0.608
		Sit-ups [30 s]	31.56 ± 4.02	30.95 ± 4.21	0.352
		Long jump [cm]	244.61 ± 19.75	237.63 ± 22.85	0.044 *
	Female	Plate tapping [s]	10.15 ± 1.36	10.28 ± 1.42	0.804
		Sit-ups [30 s]	27.29 ± 3.39	25.5 ± 3.82	0.239
		Long jump [cm]	195.11 ± 16.91	173.5 ± 17.42	0.009 *
Romania	Male	Plate tapping [s]	8.41 ± 0.84	8.61 ± 1.07	0.208
		Sit-ups [30 s]	25.65 ± 3.95	24.18 ± 3.95	0.023 *
		Long jump [cm]	232.48 ± 20.62	229.14 ± 24.29	0.377
	Female	Plate tapping [s]	9.44 ± 0.96	9.68 ± 1.1	0.606
		Sit-ups [30 s]	21.59 ± 3.81	22.71 ± 4.89	0.572
		Long jump [cm]	179.65 ± 17.07	181.86 ± 32.74	0.866

Explanations: *p*—level of statistical significance; * *p* < 0.05; BMI categories: <25—non-overweight; ≥25—overweight/obese.

**Table 4:** Multiple linear regression coefficients (β) for the associations of BMI, sex, and country with physical fitness test outcomes.

Outcome	Predictor	β	95% CI	*p*
Plate Tapping [s]	Intercept	10.417	(10.18, 10.65)	<0.001
	Sex_cat [Male]	−0.53	(−0.78, −0.28)	<0.001
	Country_cat [Romania]	−1.293	(−1.49, −1.10)	<0.001
	BMI_c	0.005	(−0.08, 0.09)	0.909
Sit-Ups [30 s]	Intercept	27.202	(26.37, 28.03)	<0.001
	Sex_cat [Male]	4.079	(3.21, 4.94)	<0.001
	Country_cat [Romania]	−6.029	(−6.72, −5.34)	<0.001
	BMI_c	−0.03	(−0.32, 0.26)	0.841
Long Jump [cm]	Intercept	191.349	(187.05, 195.64)	<0.001
	Sex_cat [Male]	53.067	(48.59, 57.54)	<0.001
	Country_cat [Romania]	−11.788	(−15.34, −8.23)	<0.001
	BMI_c	−1.614	(−3.14, −0.09)	0.038

Explanations: β—regression coefficient; CI—confidence interval; BMI_c—body mass index centered around the sample mean; Sex_cat—categorical variable (reference: female); Country_cat—categorical variable (reference: Poland). Non-significant higher-order terms (quadratic and interaction effects) were excluded from the main table for clarity and are presented in Supplementary Table S1.

**Table 5:** Model fit indices (R^2^, Adjusted R^2^, and ΔR^2^ for BMI contribution).

Outcome	R^2^	Adj. R^2^	ΔR^2^ (BMI)
Plate Tapping [s]	0.282	0.273	0.008
Sit-Ups [30 s]	0.453	0.446	0.005
Long Jump [cm]	0.599	0.594	0.011

Explanations: R^2^—coefficient of determination; Adj. R^2^—adjusted coefficient of determination; ΔR^2^ (BMI)—change in coefficient of determination reflecting the additional variance explained by BMI variables (BMI, BMI^2^, and interaction terms). Model fit indices refer to regression models including the following predictors: sex, country, centered BMI (BMI_c), squared centered BMI (BMI_c^2^), and the interaction terms BMI_c × sex and BMI_c × country.

## Data Availability

The data are not publicly available due to the presence of identifiable personal information that could compromise the privacy of research participants. However, the data are available from the corresponding author upon reasonable request.

## Supplementary Materials

The following supporting information can be downloaded at: https://www.mdpi.com/article/10.3390/sports14050167/s1, Table S1: Regression coefficients (β), 95% confidence intervals (CI), and *p*-values for BMI, BMI^2^, sex, country, and interaction terms.
