# Supplementary material for: Limited Association Between Body Mass Index and Selected Components of Physical Fitness in Higher Education Physical Education Students: A Sex- and Country-Specific Analysis
Source: Sports (Basel). 2026 Apr 22;14(5):167. doi: 10.3390/sports14050167 (PMC13210559; doi:10.3390/sports14050167)
Supplement: Supplementary file 1 [file sports-14-00167-s001.zip › sports-4232585-supplementary.pdf]

Table S1. Regression coefficients (β), 95% confidence intervals (CI), and p-values for BMI, BMI², sex, country, and interaction terms.

Outcome	Predictor	β	95% CI	p
Plate Tapping [s]	Intercept	10.417	(10.18, 10.65)	<0.001
	Sex_cat [Male]	-0.53	(-0.78, -0.28)	<0.001
	Country_cat [Romania]	-1.293	(-1.49, -1.10)	<0.001
	BMI_c	0.005	(-0.08, 0.09)	0.909
	BMI_c²	0	(-0.01, 0.01)	0.999
	BMI_c × Sex_cat [Male]	0.053	(-0.04, 0.15)	0.286
	BMI_c × Country_cat [Romania]	-0.066	(-0.13, 0.00)	0.057
Sit-Ups [30 s]	Intercept	27.202	(26.37, 28.03)	<0.001
	Sex_cat [Male]	4.079	(3.21, 4.94)	<0.001
	Country_cat [Romania]	-6.029	(-6.72, -5.34)	<0.001
	BMI_c	-0.03	(-0.32, 0.26)	0.841
	BMI_c²	0.014	(-0.02, 0.04)	0.382
	BMI_c × Sex_cat [Male]	-0.108	(-0.45, 0.23)	0.532
	BMI_c × Country_cat [Romania]	-0.061	(-0.30, 0.18)	0.616
Long Jump [cm]	Intercept	191.349	(187.05, 195.64)	<0.001
	Sex_cat [Male]	53.067	(48.59, 57.54)	<0.001
	Country_cat [Romania]	-11.788	(-15.34, -8.23)	<0.001
	BMI_c	-1.614	(-3.14, -0.09)	0.038
	BMI_c²	-0.138	(-0.30, 0.02)	0.086
	BMI_c × Sex_cat [Male]	0.686	(-1.07, 2.45)	0.444
	BMI_c × Country_cat [Romania]	0.762	(-0.47, 2.00)	0.226

Explanations: β – regression coefficient; CI – confidence interval; BMI_c – body mass index centered around the sample mean; BMI_c² – quadratic term of BMI used to test nonlinear relationships; Sex_cat – categorical variable (reference: female); Country_cat – categorical variable (reference: Poland); BMI_c × Sex_cat and BMI_c × Country_cat – interaction terms between BMI and sex and country, respectively.
